# Immunogenic Cell Death Inducers in Cancer Immunotherapy to Turn Cold Tumors into Hot Tumors

**DOI:** 10.3390/ijms26041613

**Published:** 2025-02-14

**Authors:** Valeria Lucarini, Ombretta Melaiu, Paula Gragera, Kamila Król, Valentina Scaldaferri, Verena Damiani, Adele De Ninno, Daniela Nardozi, Luca Businaro, Laura Masuelli, Roberto Bei, Loredana Cifaldi, Doriana Fruci

**Affiliations:** 1 Bambino Gesù Children’s Hospital, IRCCS, 00165 Rome, Italy; valeria.lucarini@uniroma1.it (V.L.); ombretta.melaiu@opbg.net (O.M.); paula.grageraalvarez@opbg.net (P.G.); valentina.scaldaferri@opbg.net (V.S.); verena.damiani@opbg.net (V.D.); 2 Department of Experimental Medicine, University of Rome “Sapienza”, 00161 Rome, Italy; daniela.nardozi@uniroma1.it (D.N.); laura.masuelli@uniroma1.it (L.M.); 3 Department of Clinical Sciences and Translational Medicine, University of Rome “Tor Vergata”, 00133 Rome, Italy; bei@med.uniroma2.it (R.B.); cifaldi@med.uniroma2.it (L.C.); 4 Institute for Photonics and Nanotechnologies, National Research Council, Via Fosso del Cavaliere, 00133 Rome, Italy; adele.deninno@cnr.it (A.D.N.); luca.businaro@cnr.it (L.B.)

**Keywords:** immune checkpoint inhibitors, immunogenic cell death, tumor microenvironment, tumor spheroids, neuroblastoma, anthracyclines, pediatric cancer

## Abstract

The combination of chemotherapeutic agents with immune checkpoint inhibitors (ICIs) has revolutionized cancer treatment. However, its success is often limited by insufficient immune priming in certain tumors, including pediatric malignancies. In this report, we explore clinical trials currently investigating the use of immunogenic cell death (ICD)-inducing chemotherapies in combination with ICIs for both adult and pediatric cancers. Given the limited clinical data available for pediatric tumors, we focused on recent preclinical studies evaluating the efficacy of these combinations in neuroblastoma (NB). Finally, to address this gap, we propose an innovative strategy to assess the impact of ICD-inducing chemotherapies on antitumor immune responses in NB. Using tumor spheroids derived from a transgenic NB mouse model, we validated our previous in vivo findings concerning how anthracyclines, specifically mitoxantrone and doxorubicin, significantly enhance MHC class I surface expression, stimulate IFNγ and granzyme B production by CD8^+^ T cells and NK cells, and promote immune cell recruitment. Importantly, these anthracyclines also upregulated PD-L1 expression on NB spheroids. This screening platform yielded results similar to in vivo findings, demonstrating that mitoxantrone and doxorubicin are the most potent immunomodulatory agents for NB. These data suggest that the creation of libraries of ICD inducers to be tested on tumor spheroids could reduce the number of combinations to be tested in vivo, in line with the principles of the 3Rs. Furthermore, these results highlight the potential of chemo-immunotherapy regimens to counteract the immunosuppressive tumor microenvironment in NB, paving the way for improved therapeutic strategies in pediatric cancers. They provide compelling evidence to support further clinical investigations of these combinations to enhance outcomes for children with malignancies.

## 1. Introduction

Immune checkpoint inhibitors (ICIs) have revolutionized cancer treatment and have emerged as first-line therapies for tumors with a high mutational burden or mismatch repair deficiency [1]. However, their effectiveness is often limited in malignancies characterized by insufficient immune priming and an immunologically “cold” tumor microenvironment (TME), such as pediatric tumors [2]. To address these challenges, the combination of ICIs with chemotherapy has gained significant interest due to the immunomodulatory effects of specific chemotherapeutic agents [3,4].

Recent preclinical and clinical studies have demonstrated that certain widely used chemotherapeutic agents, when administered at submaximal doses, can induce cellular stress and death, thereby stimulating adaptive antitumor immunity [5]. This process known as “immunogenic cell death” (ICD) is a regulated form of cell death that enables the initiation of adaptive immunity in immunocompetent hosts through the release of cytokines, chemokines, and damage-associated molecular patterns (DAMPs) [6,7,8,9,10]. ICD enhanced tumor sensitivity to ICIs and other forms of immunotherapy, as evidenced in several clinical trials [11,12,13,14].

DAMPs play a key role in ICD and can be divided into three main categories: prophagocytic signals, immunostimulatory molecules, and cytokines/chemokines [15]. Prophagocytic signals, such as calreticulin, heat shock protein 70 (HSP70), and HSP90, are translocated to the outer leaflet of the plasma membrane during ICD [16]. Immunostimulatory DAMPs include ATP, which is secreted via autophagy-dependent mechanisms, and nuclear or cytoplasmic proteins, such as annexin A1 (ANXA1) and high mobility group box 1 (HMGB1), which are passively released from dying tumor cells. ANXA1 facilitates dendritic cell (DC) interactions with tumor debris by binding to formyl peptide receptor 1 (FPR1) [17], while HMGB1 drives DC maturation by interacting with TLR4. Furthermore, type I IFN responses in tumor cells lead to the secretion of chemokines, such as CXC-chemokine ligand 10 (CXCL10), which promote immune cell recruitment [18].

The defining characteristics of ICDs involve four key factors: (i) the activation of stress responses leading to tumor cell death [19]; (ii) expression and presentation of tumor antigens recognized by T cells [20]; and (iii) release of DAMPs that recruit DCs, iv) promote interactions with dying tumor cells and DCs, and facilitate tumor antigen transfer. This process leads to DC maturation, migration to lymphoid organs, and antigen presentation to T lymphocytes. The fourth key point is the accessibility of DCs and T cells to tumor sites to trigger adaptive immunity and immune effector functions [19,21]. This triggers an inflammatory response leading to the recruitment and activation of antitumor immune cells [15,19]. Notably, ICD-inducing chemotherapeutics, such as anthracyclines (e.g., doxorubicin and mitoxantrone), oxaliplatin, and cyclophosphamide, can transform immunologically “cold” tumors into “hot” ones by promoting robust immune cell infiltration and tumor antigen presentation [16,17,18,22,23,24]. However, an effective anti-tumor immune response is hindered if tumors are inaccessible to T cells or if tumor cells are not immunogenic and express high levels of co-inhibitory ligands, such as PD-L1. The absence of any of these factors can lead to primary or secondary resistance to therapy.

High-risk tumors often fail to respond to ICD-inducing chemotherapeutics due to the absence of one or more of these essential factors. Therefore, combining ICD inducers with therapies that target dysfunctional immune components may enhance the efficacy of such therapy and broaden its impact. To date, the ability of ICD to sensitize tumors to ICIs has been demonstrated in several clinical trials [11,12,13,14].

In this report, we explore ongoing clinical trials evaluating combinations of ICD-inducing chemotherapeutics and ICIs in adult and pediatric cancer patients. Given the limited clinical data on pediatric tumors, we also review recent preclinical studies of combination therapies with ICD inducers and ICIs in neuroblastoma (NB). Finally, we propose a novel approach that could help identify the most effective combination therapies in a prospective manner.

### 1.1. ICD and Cancer Immunotherapy: Clinical Trials in Adult Patients

Chemotherapeutic agents that are able to induce ICD include the following: (i) anthracyclines (e.g., doxorubicin, epirubicin, idarubicin, and mitoxantrone), widely used in the treatment of solid tumors; (ii) oxaliplatin, a key component of combination regimens for colorectal cancer; (iii) cyclophosphamide, commonly employed in ovarian cancer, breast cancer, NB, and retinoblastoma; (iv) lurbinectedin, which shows potent anticancer immune responses in preclinical models [25,26,27]; (v) crizotinib, used in the treatment of metastatic solid tumors; vi) bortezomib, used primarily in multiple myeloma; and (vii) PT-112, a novel platinum–pyrophosphate conjugate [28,29,30]. In addition, some chemotherapeutic agents, while not able to robustly induce ICD, can still enhance immunogenicity under certain conditions. These include taxanes (e.g., paclitaxel, docetaxel), bleomycin, and vinca alkaloids [19,31].

Significant progress has been made in the use of combination therapies that integrate ICD-inducing agents with ICIs. For example, oxaliplatin with ICIs has been shown to convert the TME from “cold” to “hot”, promoting DC and CD8^+^ T cell infiltration and thereby sensitizing tumors to the effects of PD-1 inhibitors [3]. Conversely, regarding cisplatin, which lacks ICD-inducing properties [32], the combination of cisplatin and 5-fluorouracil has been shown to significantly enhance the release of HMGB1, promote the maturation and activation of DCs, and upregulate the co-stimulatory molecules CD80 and CD86. These effects contribute to the ICD response in tumor cells, thereby enhancing the host anti-tumor immune response and leading to a favorable prognosis [33]. This therapeutic combination not only restores the immunogenicity of cisplatin, but also reinforces the development of a robust ICD response in tumor cells.

As of October 30, 2024, 357 active clinical trials listed on ClinicalTrials.gov (https://clinicaltrials.gov) (initiated after 1 January 2020) are evaluating ICD-inducing chemotherapy in combination with FDA-approved ICIs. Among these trials, paclitaxel is the most commonly used ICD-inducing agent, followed by docetaxel, cyclophosphamide, oxaliplatin, and doxorubicin. The most commonly paired ICIs include anti-PD-1 agents (e.g., pembrolizumab, nivolumab and dostarlimab), anti-PD-L1 agents (e.g., atezolizumab, durvalumab, and avelumab), and, to a lesser extent, anti-CTLA4 agents (e.g., ipilimumab). Interestingly, tumor-specific combinations have been identified. For example, paclitaxel and docetaxel are often combined with ICIs in non-small cell lung cancer. Oxaliplatin-based combinations are mainly used in gastric and colorectal cancer, while cyclophosphamide and doxorubicin combinations are used in breast cancer.

Combination regimens of ICD inducers and ICIs have demonstrated promising results in several cancer types. For example, in triple-negative breast cancer (TNBC), a combination of doxorubicin and nivolumab (PD-1 inhibitor) achieved an objective response rate (ORR) of 35% (TONIC Trial) [11]. Similarly, low-dose doxorubicin and cyclophosphamide plus atezolizumab (PD-L1 inhibitor) improved survival in patients with TNBC (NCT03164993) [14]. Atezolizumab in combination with nab-paclitaxel and doxorubicin significantly increased pathological complete response rates in TNBC patients with acceptable safety profiles (IMpassion031 trial) [34]. Furthermore, in recurrent ovarian cancer, a phase 2 trial of pembrolizumab in combination with bevacizumab and low-dose cyclophosphamide showed clinical benefit in 95% of patients, of which 25% showed durable responses (>12 months) (NCT02853318) [35]. Moreover, in the TONIC trial, the combination of nivolumab with doxorubicin resulted in the highest ORR (35%), compared to nivolumab with cisplatin (23%), highlighting the enhanced efficacy of ICD-inducing agents in combination with ICIs [11]. These findings highlight the transformative potential of combining ICD-inducing chemotherapies with immunotherapy to enhance treatment efficacy and broaden the therapeutic scope across multiple cancers.

### 1.2. ICD and Cancer Immunotherapy in Pediatric Cancers: Preclinical Studies

While immunotherapy has revolutionized cancer treatment in adults, its application in pediatric malignancies remains underexplored. Although several immune checkpoint molecules have been investigated for their prognostic value [36], their functional roles and potential as therapeutic targets in pediatric cancers are poorly understood, and data from preclinical studies are limited [36,37,38,39].

The few immunotherapy studies in pediatric tumors, such as PD-1 blockade, have generally shown limited efficacy. This includes NB, the most common extracranial pediatric solid tumor and the leading cause of cancer-related death in children [40]. Despite intensive treatment, children with refractory or relapsed NB have a poor prognosis, with event-free survival rates below 10% [40]. High-risk NB represents a significant therapeutic challenge due to its immunologically “cold” TME, characterized by limited immune cell infiltration [41,42], tumor heterogeneity [41], low mutational burden, and the reduced expression of MHC class I molecules and antigen processing and presentation (APP) pathway components, which limit tumor antigen recognition by T cells [2,43,44,45,46]. These characteristics make NB an ideal model for studying the barriers to effective antitumor immune responses.

Recent evidence, including our own, suggests that combining ICD-inducing chemotherapeutic agents with immunotherapy can effectively sensitize poorly T cell-infiltrated NB to the host antitumor immune response. This dual approach not only promotes the recruitment of immune cells into the tumor, but also targets the immunosuppressive factors in the TME [47,48]. For example, Webb and colleagues have shown that cyclophosphamide selectively depletes intratumoral regulatory T (Treg) cells in NB mouse models, and when combined with anti-PD-1 therapy, this approach significantly improves tumor control [48]. In this context, we have recently shown that combining ICD-inducing chemotherapy with therapies targeting missing immunomodulatory factors can enhance outcomes in NB [47]. We developed an in situ immunomodulation strategy that combines (i) low-dose chemotherapy to induce ICD and recruit immune cells into the TME, with (ii) anti-TGFβ therapy to mitigate immunosuppressive mechanisms in the TME and (iii) anti-PD-1 therapy to restore the functionality of tumor-infiltrating immune cells [47]. This approach was evaluated through ex vivo analyses of murine and patient-derived NB tissues and in vivo studies using mouse models of NB grown subcutaneously or orthotopically in the adrenal gland. The results identified a novel combination therapy based on mitoxantrone, anti-TGFβ, and PD-1 blockade. This regimen significantly impaired tumor progression, induced a substantial regression of tumor growth, and remodeled the tumor immune landscape [47]. We observed the recruitment of diverse innate and adaptive immune cells into the TME, including DCs and effector CD8^+^ T cells and NK cells, both of which expressed IFNγ and granzyme. These immune responses were accompanied by the increased production of inflammatory chemokines, such as CCL5, CXCL9, and CXCL10, which are critical for the recruitment of tumor-specific CD8^+^ T cells and functional DC and NK cells, which are associated with improved survival in NB patients [45,49]. In addition, we have shown that this treatment is also efficacious in patient-derived spheroids derived from human NB specimens [50], providing a proof of principle for the potential of the proposed combinatorial strategy to recruit and activate CD8^+^ T cells and NK cells in a patient-specific context [47]. Therefore, further investigation in a prospective study will be critical to confirm the therapeutic efficacy of mitoxantrone treatment in NB.

## 2. Results and Discussion

### 3D Tumor Spheroid Models: Advancing the Study of Immunomodulatory Agents in the Pediatric Tumor Microenvironment

The evaluation of the efficacy of immunomodulatory agents designed to recruit immune cells into the TME requires advanced cellular models that closely mimic tumor–immune interactions [50]. Three-dimensional (3D) tumor spheroid models have emerged as valuable tools, offering advantages over traditional two-dimensional (2D) cultures by better mimicking the spatial organization, cell interactions, and gradients of oxygen, nutrients, and signaling molecules found in the TME [51].

The integration of immune cells into syngeneic 3D spheroid systems allows for a more accurate study of how immunomodulatory agents influence immune cell infiltration, activation, and function in a tumor-like environment [52]. These models provide a physiologically relevant platform for preclinical drug screening, improving the evaluation of therapeutic efficacy and advancing the development of immunotherapies.

Given the wide range of potential combinations between ICD inducers and ICIs, the use of a screening platform to identify the most effective ICD inducers for combination with immunotherapy could optimize therapeutic strategies for pediatric malignancies. Therefore, as a proof of principle, we evaluated the suitability of 3D NB models for screening immunomodulatory drugs by generating tumor spheroids using the NB murine cell lines 9475A2 and 9464D, which were also used in the in vivo study [47]. The spheroids were treated with the same compounds tested in vivo, ensuring consistency between experimental approaches [47]. This setup allowed for a comparative analysis of drug efficacy in a controlled tumor mimicking environment.

**Figure 1:**
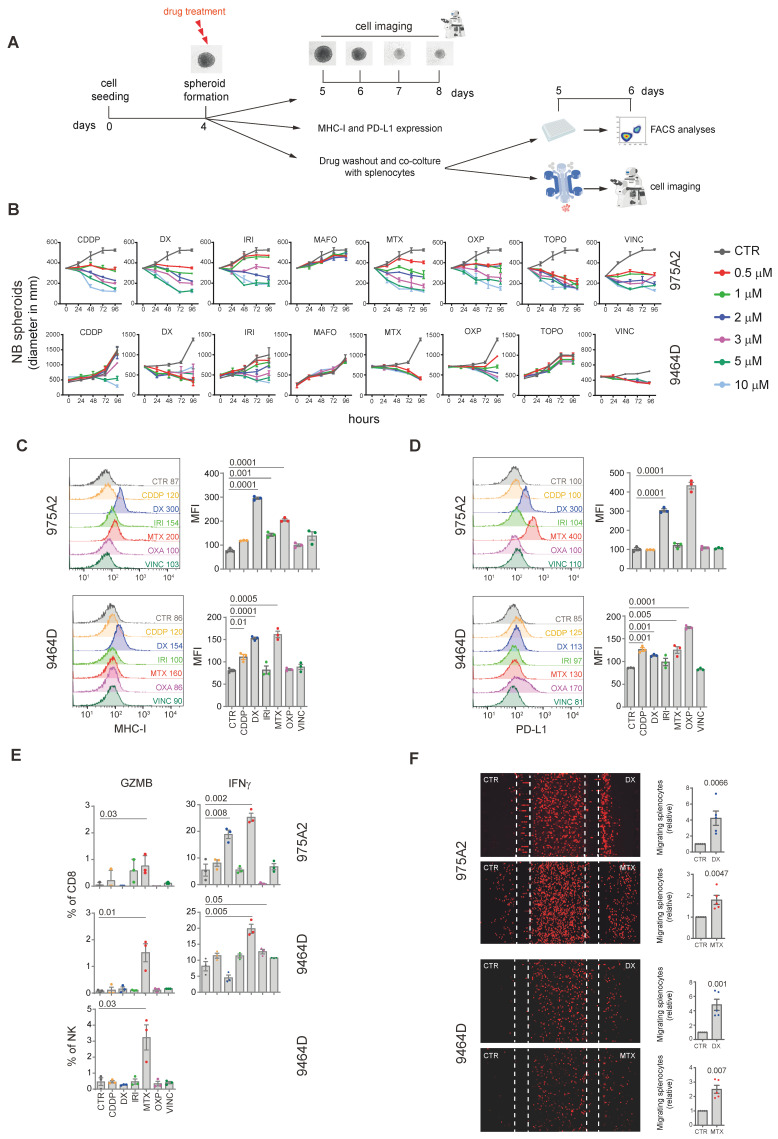
Chemotherapeutic drugs promote the recruitment and activation of CD8^+^ T cells and NK cells in co-cultures of NB spheroids and syngeneic splenocytes. (**A**) Experimental scheme. NB cells were seeded in ULA plates to form spheroids. The 4-day-old NB spheroids were either treated with the indicated compounds at different concentrations while monitoring their diameter over the next 96 hours or treated for 1 day, and then, after drug removal, were cultured with syngeneic splenocytes in ULA plates or a microfluidic device. (**B**) Time–course and dose–response curves of drug-treated 9464D and 975A2 NB spheroids. Spheroid’s diameter was evaluated from 0 to 96 hours. Each color corresponds to a tested concentration (e.g., red for 0.5 µM, green for 1 µM, etc.). Spheroid diameter was analyzed using ImageJ software v1.54j, NIH Image, NIH, Bethesda, MD, USA). Data are the mean ± SEM of 3 independent experiments in triplicate. (**C**,**D**) Representative flow cytometric analyses of MHC class I (**C**) and PD-L1 (**D**) cell surface expression in NB spheroids treated with the indicated drugs for 24 hours. The graphs represent the MFI ± SD of three independent experiments. Significance levels for comparison between samples were determined by two-tailed Student’s *t* test. (**E**) Flow cytometric analysis of IFNγ and granzyme B expression of CD8^+^ T cells and NK cells from splenocytes co-cultured with drug-treated NB spheroids for 24 hours. Significance levels for comparison between samples were determined by ANOVA. (**F**) Representative images of migration in microfluidic devices of red-labeled splenocytes recruited from drug-treated and untreated NB spheroids after 24 hours of co-culture (**left**). The number of splenocytes migrating toward treated and untreated NB spheroids is assessed using ImageJ software v1.54j, NIH Image, NIH, Bethesda, MD, USA). Data are shown as fold change ± SD (**right**). Significance levels for comparison between samples were determined by Student’s *t*-test. CTR, control; CDDP, cisplatin; DX, doxorubicin; IRI, irinotecan; MAFO, mafosfamide; MTX, mitoxantrone; OXP, oxaliplatin; TOPO, topotecan; VINC, vincristine; GZMB, granzyme B; IFNγ, interferon gamma.

We first assessed the effect of drugs on spheroid growth by measuring spheroid diameters at increasing concentrations (Figure 1A). All compounds except mafosfamide inhibited the growth of 975A2 spheroids in a concentration-dependent manner (Figure 1B). Similar results were observed for 9464D spheroids, except for topotecan and mafosfamide, which had no effect (Figure 1B). Drugs that inhibited spheroid growth were further tested at a metronomic dose.

We then evaluated the effects of these drugs on the surface expression of MHC class I and PD-L1 molecules on the 9464D and 975A2 cell lines, which endogenously express low levels of MHC-I and significant levels of PD-L1 [46,47]. Doxorubicin and mitoxantrone significantly increased MHC class I expression in both 975A2 and 9464D spheroids (Figure 1C). Similar effects were observed for irinotecan and cisplatin in 975A2 and 9464D spheroids, respectively (Figure 1C). Interestingly, doxorubicin and mitoxantrone also significantly increased PD-L1 expression in both spheroid models, whereas cisplatin and oxaliplatin increased PD-L1 expression in 9464D spheroids (Figure 1D).

The immunomodulatory effects of the selected compounds were further analyzed in co-culture experiments using drug-treated and untreated spheroids and splenocytes from tumor-bearing mice (Figure 1A). We devised multi-color flow cytometry panels and progressive gating strategies to analyze tumor-infiltrating lymphoid cell populations (Figure S1). Flow cytometry revealed that mitoxantrone treatment significantly enhanced IFNγ and granzyme B production by CD8^+^ T cells in both 975A2 and 9464D spheroids (Figure 1E). Mitoxantrone-treated 9464D spheroids also significantly promoted granzyme B production by NK cells (Figure 1E). Furthermore, although not significant, an increase in granzyme B and IFNγ production by NK cells was induced by mitoxantrone-treated 975A2 and 9464D spheroids, respectively (Figure 1E). Doxorubicin and oxaliplatin treatments further increased IFNγ production by CD8^+^ T cells in 975A2 and 9464D spheroids, respectively (Figure 1E). In addition, the effects of these chemotherapeutic agents on DCs were analyzed. Specifically, we observed an increase in cDC1 following treatment with DX and MTX (Supplementary Figure S1).

Finally, to study the dynamic interactions between immune and tumor cells, we used microfluidic devices to assess immune cell recruitment (Figure 1A). Treatment with mitoxantrone and doxorubicin significantly enhanced immune cell migration towards both 975A2 and 9464D spheroids compared to untreated controls (Figure 1F). In contrast, no migration was observed following CDDP treatment (Supplementary Figure S1).

Collectively, these results demonstrate that mitoxantrone and doxorubicin are the most effective anthracyclines for enhancing the immunogenicity of NB spheroids. These agents upregulated MHC class I expression on NB spheroids, a critical mechanism for enhancing tumor cell recognition by CD8^+^ T cells. This improved immune surveillance facilitates effective effector functions and the recruitment of CD8^+^ T cells and NK cells, suggesting their potential to convert a “cold” TME into a “hot” one. This highlights the therapeutic potential of these drugs in NB treatment by enhancing immune cell infiltration and activation within the TME. In addition, the induction of PD-L1 expression following drug treatment justifies the use of PD-L1 inhibitors that could potentiate the antitumor immune response and underscores the need to combine chemotherapy with ICIs to counteract such resistance and maximize therapeutic outcomes.

Mitoxantrone, already approved for cancers, such as acute non-lymphoblastic leukemia and advanced prostate cancer, showed remarkable efficacy in high-risk NB by enhancing anti-tumor immune responses when combined with ICIs [47].

## 3. Material and Methods

### 3.1. Mice, Cell Lines and Reagents

Six- to eight-week-old female C57BL/6 mice (Charles River Laboratories, Wilmington, MA, USA) were housed under pathogen-free conditions in the Plaisant (Rome, Italy) animal facility, and experiments were performed in accordance with the 3Rs policy (authorization of Italian Ministry of Health n. 755/2019-PR). Transgenic NB cell lines 9464D and 975A2, derived from spontaneous tumors arising in TH-MYCN transgenic mice on a C57BL/6 background, were kindly provided by Dr Crystal Mackall (Stanford University, Stanford, CA, USA). Tumor cells were cultured in RPMI with 10% FCS with Pen/Strep/Glut at 37 °C and 5% CO2 on tissue-culture treated plastic plates. Doxorubicin hydrochloride, mitoxantrone dihydrochloride, and oxaliplatin were from Sigma-Aldrich St. Louis, Missouri, USA). Cisplatin (Accord Healthcare Limited, London, UK), vincristine (Pfizer), irinotecan (Campo, Pfizer, Latina, Italy), mafosfamide (4-sulfoethylthio- cyclophosphamide L-lysine; Niomech-IIT GmbH), and topotecan (GlaxoSmithKline, Brentford, UK) were provided by the pharmacy of the Bambino Gesù Children’s Hospital (Rome, Italy).

### 3.2. Tumor Spheroid Growth, Drug Treatment, and Co-Culture Experiments

Tumor spheroids were generated by plating 200 μL/well of 9464D or 975A2 cells at the optimized densities of 1 × 10^4^ cells/mL in 96-well round-bottom ULA plates (Corning B.V. Life Sciences, Amsterdam, The Netherlands). Plates were incubated at 37 °C, 5% CO2, and 95% humidity for 4 days and then treated with the indicated drugs at concentrations ranging from 0.5 to 10 µM. Control spheroids were treated with the appropriate vehicles. Responses to drug treatment were assessed by measuring the diameter of the spheroids at regular intervals of 24, 48, 72, and 96 h from images captured with an inverted microscope (LEICA DMi8) equipped with a CCD camera (Hamamatsu ORCA-Flash4.0 LT + Digital CMSO camera C11440-42U30) and ImageJ software v1.54j analysis (http://imagej.net/ij), as previously described. In co-culture experiments, drug-treated and drug-untreated spheroids were co-cultured with splenocytes from naïve C57BL/6 mice for 24 h, and the different immune cell populations were analyzed by flow cytometry using BD LSR Fortessa X20 with FACSDiva Software v9.0 (BD Bioscences, Franklin Lakes, NJ, USA) and FlowJo software (version 10.7.2).

### 3.3. Flow-Cytometry

All antibodies for flow cytometry analysis were purchased from BD Pharmingen (Franklin Lakes, NJ, USA), eBioscience (San Diego, CA, USA), Invitrogen (Thermo Fischer Scientific Inc., Waltham, MA, USA), and Biolegend (San Diego, CA, USA) [45]. For surface staining, cells were stained with fluorescent-labeled antibodies. For intracellular staining, cells were seeded at a density of 1 × 10^6^ cells per well in 96-well U-bottomed plates and stained with antibodies targeting surface markers. The cells were then fixed with 2% PFA for 10 min at 25 °C, permeabilized using 0.2% Saponin, and subsequently stained with anti-IFNγ and anti-granzyme B antibodies, following the Fixation/Permeabilization Concentrate and Diluent kit (eBioscience) instructions. Samples were analyzed using a BD Fortessa flow cytometer, and data were processed using FlowJo software.

### 3.4. Microfluidic Device Migration Assay

The immunomodulatory effects of drugs were evaluated with microfluidic devices fabricated from polydimethylsiloxane, a biocompatible silicone elastomer, following a standard replica molding technique [50]. Prior to cell introduction, the devices were sterilized under UV light for 30 min. NB spheroids were suspended in Matrigel (2 mg/mL; BD Biosciences) and exposed to drugs at the specified concentrations. Both drug-treated and untreated spheroids were placed into the side chambers of the devices (1 × 10^4^ cells in 3 μL), separated from the central chamber by microchannels, and incubated at 37 °C for 30 min to allow the gel to solidify. Subsequently, 1 × 10^6^ splenocytes from tumor-bearing C57BL/6 mice were labeled with Cell Tracker Red (Invitrogen), suspended in complete RPMI medium, and added to the central chamber. The microchannel design allowed splenocytes to migrate into the side chambers while preventing spheroids from entering the central chamber. Bright-field and fluorescence images were taken using a LEICA DMi8 microscope 24 h after loading. The migration of splenocytes was calculated by counting the red-labeled cells in the side chambers using ImageJ software v1.54j (http://imagej.netij).

### 3.5. Statistical Analysis

Statistical analysis was performed using GraphPad Prism 8.0.2 software to determine the significance between samples. The statistical tests used are specified in the figure legends. Unless otherwise noted, all data are representative of at least three independent experiments. Error bars represent the standard error of the mean (SEM) and are based on triplicate experimental conditions. A *p*-value of ≤0.05 was considered significant.

## 4. Conclusions

In conclusion, we have attempted to highlight the transformative potential of combining ICD-inducing chemotherapeutic agents with immunotherapy to improve treatment efficacy, particularly for tumors such as high-risk NB that are poorly infiltrated by immune cells and typically resistant to immunotherapy. The ability of chemotherapeutic agents such as mitoxantrone and doxorubicin to modulate the immune response and promote immune cell recruitment offers a promising strategy to overcome the immunosuppressive TME. By shifting the TME from “cold” to “hot”, these therapies facilitate immune cell infiltration and activate effector molecules critical for the anti-tumor response.

We have also demonstrated that 3D tumor spheroid models can be superimposed on in vivo syngeneic models to study the effects of immunomodulatory agents on immune cell infiltration, activation, and function. This provides the basis for the development of platforms for the preclinical screening of these drugs and new immunotherapies. Indeed, the use of automated imaging platforms that allow for the serial imaging of spheroids co-cultured with immune cells could facilitate the screening of a much wider range of drugs than we have explored to date. In addition, the integration of artificial intelligence or machine learning could further improve the analysis of the large datasets generated by these platforms, increasing the efficiency and scope of these studies.

These results support ongoing clinical trials investigating chemo-immunotherapy combinations for pediatric cancers and highlight the need for further research to assess their long-term benefits. Ultimately, combining ICD-inducing chemotherapies with immunotherapy may provide a more effective treatment approach, expanding the therapeutic scope for various cancers and improving outcomes for patients with hard-to-treat malignancies.

## Data Availability

Data is contained within the article and Supplementary Materials.

## Supplementary Materials

The following supporting information can be downloaded at https://www.mdpi.com/article/10.3390/ijms26041613/s1.
