# Supplementary material for: Immunogenic Cell Death Inducers in Cancer Immunotherapy to Turn Cold Tumors into Hot Tumors
Source: Int J Mol Sci. 2025 Feb 14;26(4):1613. doi: 10.3390/ijms26041613 (PMC11855819; doi:10.3390/ijms26041613)
Supplement: Supplementary file 1 [file ijms-26-01613-s001.zip › ijms-3431737-supplementary.pdf]

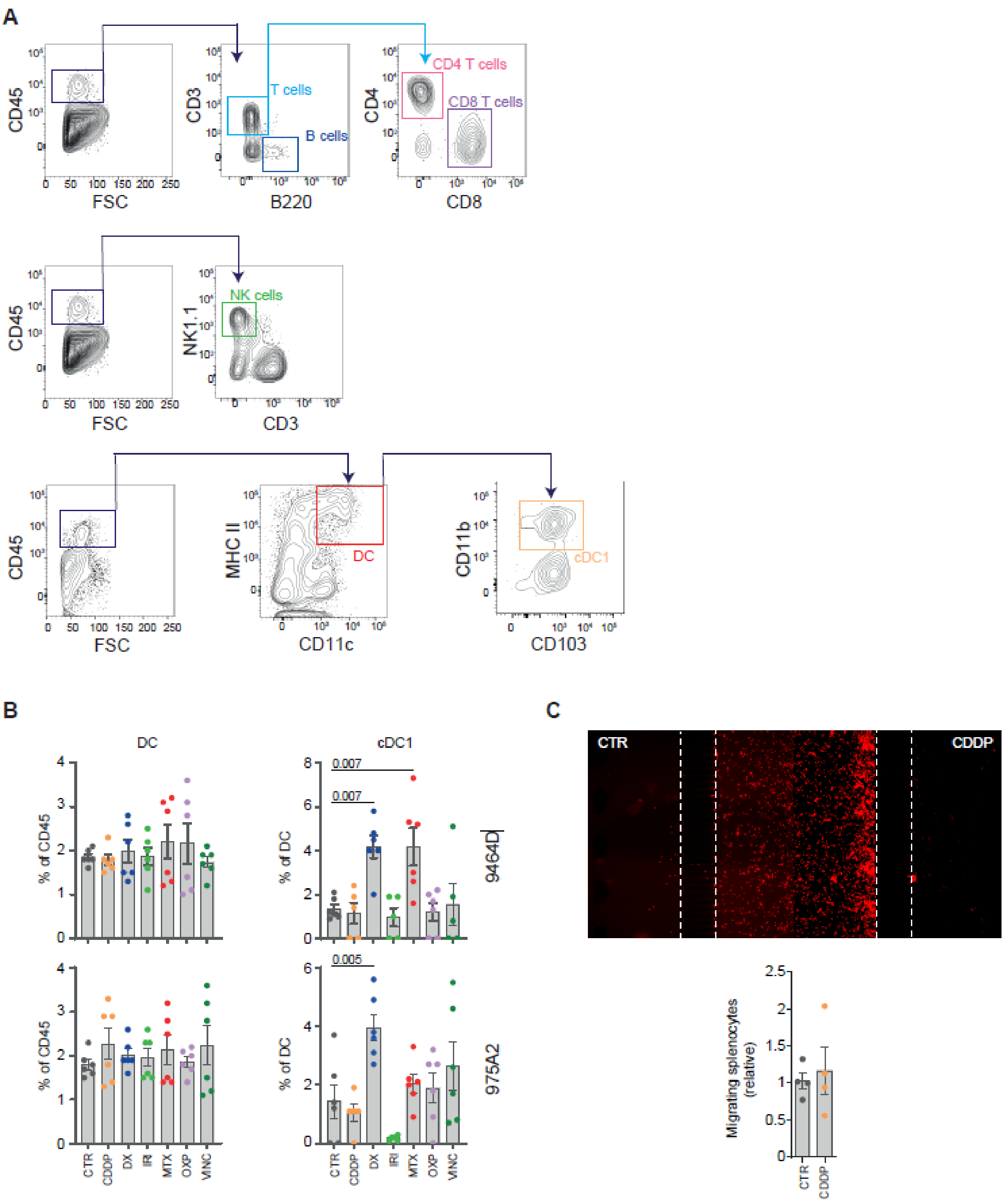

Figure S1. A) Representative flow cytometric gating strategy for NB spheroids to define tumor-infiltrating lymphoid cells. B) Flow cytometry analysis of DC and cDC1 cells from splenocytes co-cultured with drug-treated NB spheroids for 24 hours. Significant levels for comparison between samples were determined by ANOVA. C) Representative images of migration in microfluidic devices of red-labeled splenocytes recruited from cisplatin-treated and untreated NB spheroids after 24 hours of co-culture. The number of splenocytes migrating towards treated and untreated NB spheroids was assessed using ImageJ software. Data are shown as fold change ± SD.
